# Supplementary material for: Surface Microbiome Profiling of Dental Elevators Using Third‐Generation Sequencing: Implications for Infection Control in Dental Practice
Source: Microbiologyopen. 2025 Dec 8;14(6):e70178. doi: 10.1002/mbo3.70178 (PMC12685768; doi:10.1002/mbo3.70178)
Supplement: Supplementary file 1 — customer_backup. [file MBO3-14-e70178-s001.zip › customer_backup/customer_backup/taxa_summary/krona/samples/M01.Krona.html]

Javascript must be enabled to view this page.

members
magnitude
magnitudeUnassigned

M01.krona

55481

55477

213

213

213

213

213

2

9

54

27

49

28

6

38

10438

430

430

1

1

1

429

50

48

2

9

9

69

69

174

13

160

1

127

3

123

1

156

156

156

156

156

1238

215

62

35

35

25

25

2

2

153

153

153

194

33

33

33

10

10

10

9

9

9

21

21

21

87

87

87

34

34

34

37

37

37

37

792

2

2

1

1

145

145

1

11

133

494

34

21

13

445

25

420

15

2

13

151

14

14

137

137

6048

4332

4

4

4

1642

1303

5

586

204

41

389

78

24

2

22

255

30

18

207

13

10

3

47

47

2686

68

68

2593

2593

7

7

18

18

1716

1113

1113

183

885

45

603

16

16

24

24

563

563

2324

1

1

1

1

19

19

19

19

28

28

1

1

27

2

24

1

151

151

5

5

3

3

15

6

9

10

10

11

11

43

9

2

3

29

29

25

4

18

18

1

1

16

16

541

293

45

45

14

2

12

1

1

233

233

248

29

23

6

166

43

29

15

2

2

10

11

8

1

9

11

1

24

39

39

14

14

3

3

3

3

109

109

109

19

70

19

1

159

1

1

1

135

135

34

34

19

48

23

20

3

17

1

1

2

2

152

18

16

16

2

2

13

13

13

13

13

13

20

20

20

22

22

3

19

66

6

6

1

1

59

59

416

416

12

12

363

3

39

21

54

166

80

10

10

27

13

7

4

3

1

1

3

3

683

202

9

9

6

6

1

1

186

13

173

172

1

1

9

9

1

1

3

3

89

9

55

6

19

4

4

15

15

50

50

28

27

26

1

1

1

265

4

4

1

1

2

2

47

1

46

32

32

5

5

19

19

4

4

137

4

23

110

9

9

4

4

1

1

1

1

1

15

15

15

62

5

4

4

1

1

57

1

1

56

56

242

242

242

242

5

134

99

4

5

4

4

4

4

1

3

1

1

1

1

1

440

440

440

440

440

440

87

87

87

86

13

13

73

1

72

1

1

1

90

90

90

89

89

63

26

1

1

1

5

5

5

1

1

1

4

4

4

1918

1802

3

3

3

3

12

12

2

2

10

10

1787

2

1

1

1

1

1785

1784

1784

1

1

116

116

5

5

5

22

22

22

5

5

5

16

16

16

68

68

68

38

38

38

38

1

1

37

37

305

267

267

267

166

164

2

81

26

51

4

20

20

38

26

26

26

26

12

12

12

12

17

17

17

1

1

1

2

2

2

2

2

2

1

1

1

3

3

3

8

8

8

1229

687

57

57

57

57

630

170

130

96

34

2

2

28

28

10

10

460

10

10

109

109

12

12

329

329

343

302

302

302

56

199

40

7

8

8

8

8

33

33

9

9

8

1

1

6

4

2

2

8

8

4

3

1

17

3

3

3

3

14

14

14

14

98

98

98

98

3

1

12

2

7

73

84

84

13

13

13

5

5

5

66

66

66

3

3

3

3

3

3

3

3

3

3

3

3

423

3

3

3

3

3

4

4

4

2

2

1

1

1

1

416

416

416

41

28

13

366

1

130

235

8

8

1

1

1

1

1

1

1

1

1025

252

252

252

252

252

773

536

448

426

113

237

76

22

15

6

1

88

88

87

1

12

12

12

12

196

10

2

2

8

8

2

2

2

184

184

62

122

29

29

3

3

26

7

19

1

1

1

1

1

1

126

42

2

2

1

1

1

1

40

32

32

32

3

3

3

1

1

1

3

3

3

1

1

1

26

26

26

26

26

58

58

58

58

58

18038

6532

104

6

6

6

97

1

1

96

1

95

1

1

1

490

490

240

240

246

246

4

4

73

73

22

22

49

49

2

2

135

135

13

5

7

1

122

4

5

1

112

685

685

18

1

3

1

1

12

2

2

1

1

93

93

29

2

4

9

5

1

4

4

106

12

49

26

19

4

4

360

22

57

6

21

51

1

96

5

3

5

1

55

23

1

13

72

46

2

24

6

6

6

6

4459

158

68

1

67

2

2

6

3

3

82

28

22

32

26

26

26

69

38

16

1

4

1

7

9

12

4

8

19

19

12

12

12

15

15

15

1

1

1

13

13

13

237

39

1

5

33

3

3

195

41

137

2

15

24

24

24

194

142

142

52

52

1526

35

12

23

158

158

448

448

13

11

2

4

4

4

4

9

9

855

58

797

1138

368

337

31

2

2

10

10

79

79

535

2

375

31

87

33

7

1

1

143

3

3

36

27

32

2

10

3

15

12

134

10

10

1

1

53

53

70

70

138

138

1

137

774

49

49

32

2

30

3

3

20

12

3

4

1

3

3

40

40

576

47

169

7

124

2

2

190

2

33

51

51

94

25

9

9

16

16

69

7

7

46

3

11

7

25

10

1

3

5

1

2

2

4

1

3

11

11

11

11

1

1

1

1

10

10

10

10

20

20

20

17

3

2

2

2

2

255

255

17

17

90

90

19

2

1

7

1

8

70

10

52

8

38

3

27

8

4

4

17

17

37

9

9

9

2

2

2

2

2

2

6

6

6

2

2

2

1

1

1

8

8

8

7

7

7

150

150

44

31

13

2

2

4

3

1

71

20

51

29

12

9

8

11506

47

43

43

43

4

4

4

2064

3

2

2

1

1

1

1

1

5

5

5

1939

1826

1826

12

12

81

81

20

20

3

3

2

1

101

101

101

5

5

5

6

6

6

1

1

1

8591

1

1

1

20

1

1

19

19

11

11

11

14

14

14

64

64

64

102

99

99

3

3

2402

6

6

7

7

2161

2161

188

13

90

85

40

2

1

1

4

1

1

4

2

14

10

102

4

4

28

28

42

5

15

22

3

3

20

20

5

5

3

3

3

36

1

1

3

3

3

3

1

1

28

28

77

1

1

76

76

135

135

135

5485

4

3

1

20

20

139

139

17

1

16

134

2

42

90

12

1

11

38

33

5

11

11

384

33

351

27

27

17

17

4677

4677

5

5

139

84

84

55

44

11

246

219

140

124

16

68

68

11

11

27

27

26

1

150

150

139

11

93

35

11

11

36

36

36

36

58

58

58

58

5

5

5

2

3

194

37

2

2

4

4

3

3

11

11

17

3

14

157

17

17

4

4

15

15

71

10

1

60

50

4

3

3

6

1

32

1

1

1

1

1

1

1

1

1

113

25

25

25

73

73

2

67

4

15

1

1

1

1

13

1

12

533

125

125

125

6

6

4

4

1

1

2

2

19

19

53

53

40

38

2

408

41

41

41

41

1

1

1

1

173

65

9

4

5

9

9

8

1

7

39

9

30

90

1

1

89

64

25

18

6

6

12

12

193

193

193

5

19

91

78

4

4

4

4

4

1

2

1

151

14

13

13

13

13

1

1

1

1

11

10

10

10

10

1

1

1

1

45

1

1

1

1

4

4

4

4

3

3

3

3

12

12

12

12

25

24

24

24

1

1

1

81

81

81

81

81

15589

7598

29

29

6

6

2

2

21

3

1

1

16

19

19

19

19

133

1

1

1

6

6

6

126

126

126

40

40

40

7

33

5440

5435

2

2

39

2

37

291

5

166

8

39

8

1

11

29

13

6

4

1

117

16

101

14

14

64

48

16

1604

666

1

933

4

154

145

9

420

420

8

8

534

71

1

458

4

141

141

58

58

4

4

72

72

46

46

266

3

121

142

15

15

146

146

1

1

31

31

43

31

12

3

3

109

36

73

3

3

1231

28

809

5

32

8

99

114

15

4

64

35

18

19

15

4

5

5

5

342

224

62

37

25

1

1

143

69

74

18

18

4

4

4

112

3

3

1

1

15

15

93

71

22

2

1

1

1

1

33

33

31

3

1

13

14

2

2

16

4

4

4

1

1

1

11

11

11

3

3

3

3

150

20

20

20

130

130

130

1393

1

1

1

684

18

18

27

27

45

33

5

3

4

61

2

56

3

268

268

1

1

39

1

1

13

24

8

8

33

33

1

1

25

25

5

5

153

153

179

179

179

8

1

1

7

7

482

1

1

1

1

120

42

29

14

35

198

198

107

12

5

3

87

40

40

15

9

6

36

7

7

16

15

1

13

13

3

3

3

7923

2684

2074

1

1

2073

28

2

33

110

1703

48

45

3

75

26

610

14

14

18

18

7

7

99

80

2

6

2

9

262

9

253

11

11

113

113

86

86

1

1

1

1

1931

407

119

119

208

131

74

3

80

52

28

1524

617

598

19

10

10

6

6

245

245

1

1

128

7

121

241

241

37

37

138

27

111

28

1

27

50

50

23

23

757

756

1

1

755

4

751

1

1

1

365

365

298

8

8

7

1

7

3

13

20

9

10

171

1

40

14

14

53

44

9

6

6

6

6

779

779

779

775

4

30

13

13

13

7

7

7

10

10

10

1369

6

6

2

4

71

2

2

68

68

1

1

145

139

15

1

16

2

101

4

6

6

30

30

1

29

1116

1

1

10

8

2

5

3

2

312

1

197

114

7

7

492

414

78

21

21

245

245

11

6

5

4

4

8

6

2

1

1

1

1

1

1

1

6

6

6

6

6

62

62

9

9

9

20

20

20

12

1

1

11

10

1

21

21

21

38

38

38

38

38

35

3

3

3

3

3

3

2

1

232

147

5

5

5

5

142

141

141

141

1

1

1

85

85

85

50

39

11

24

24

11

7

4

4093

4087

506

11

11

11

495

2

2

31

10

21

63

33

1

24

5

115

5

106

4

47

47

1

1

44

11

5

1

1

14

7

5

72

9

32

13

18

3

3

39

39

71

2

69

3

3

4

4

67

46

21

21

12

12

13

1

12

8

8

8

13

13

13

3350

271

5

5

59

59

207

19

3

3

2

70

27

83

253

6

6

247

17

34

196

1

1

1

989

989

1

6

53

788

22

4

2

112

1

518

95

95

423

13

127

90

97

2

94

193

86

86

50

50

55

55

1

1

1

1

11

11

11

1114

8

8

44

44

356

356

706

706

6

6

4

3

1

2

2

158

16

16

16

1

1

1

24

1

1

23

5

18

42

42

42

22

6

1

2

3

11

11

1

1

4

1

1

1

1

53

14

14

27

27

12

10

2

1

1

1

1

1

5

5

5

5

5

404

404

404

44

44

44

86

86

86

274

274

274

25

25

25

25

25

25

4

4

4

4

4

1

1

3

3
